# Stable and Local Reservoirs of *Mycobacterium ulcerans* Inferred from the Nonrandom Distribution of Bacterial Genotypes, Benin

**DOI:** 10.3201/eid2603.190573

**Published:** 2020-03

**Authors:** Clément Coudereau, Alban Besnard, Marie Robbe-Saule, Céline Bris, Marie Kempf, Roch Christian Johnson, Télésphore Yao Brou, Ronald Gnimavo, Sara Eyangoh, Fida Khater, Estelle Marion

**Affiliations:** Université d’Angers, Angers, France (C. Coudereau, A. Besnard, M. Robbe-Saule, M. Kempf, F. Khater, E. Marion);; INSERM, Angers (C. Coudereau, A. Besnard, M. Robbe-Saule, M. Kempf, F. Khater, E. Marion);; Centre Hospitalo-Universitaire d’Angers, Angers (C. Bris, M. Kempf);; Université d'Abomey Calavi, Abomey Calavi, Benin (R.C. Johnson);; Fondation Raoul Follereau, Paris, France (R.C. Johnson);; Maison de la Télédétection, Montpellier, France (T.Y. Brou);; Centre de Diagnostic et Traitement de la Lèpre et de l’Ulcère de Buruli, Pobè, Bénin (R. Gnimavo);; International Pasteur Institute Network, Yaoundé, Cameroon (S. Eyangoh)

**Keywords:** Mycobacterium ulcerans, Buruli ulcer, genetic diversity, mutational profiling, genomic analysis, geospatial model, genotyping, tuberculosis and other mycobacteria, Benin, Nigeria, bacteria

## Abstract

*Mycobacterium ulcerans* is the causative agent of Buruli ulcer, a neglected tropical disease found in rural areas of West and Central Africa. Despite the ongoing efforts to tackle Buruli ulcer epidemics, the environmental reservoir of its pathogen remains elusive, underscoring the need for new approaches to improving disease prevention and management. In our study, we implemented a local-scale spatial clustering model and deciphered the genetic diversity of the bacteria in a small area of Benin where Buruli ulcer is endemic. Using 179 strain samples from West Africa, we conducted a phylogeographic analysis combining whole-genome sequencing with spatial scan statistics. The 8 distinct genotypes we identified were by no means randomly spread over the studied area. Instead, they were divided into 3 different geographic clusters, associated with landscape characteristics. Our results highlight the ability of *M. ulcerans* to evolve independently and differentially depending on location in a specific ecologic reservoir.

Buruli ulcer (BU) is a devastating necrotic human skin disease caused by *Mycobacterium ulcerans* (*1*). It is the third most common mycobacterial disease after tuberculosis and leprosy; ≈2,000 cases are reported each year worldwide, mostly in rural areas of West and Central Africa. The high number of patients with massive skin ulcers is a major problem because treatment of advanced disease is complex, and the consequent long-term disabilities can lead to social stigmatization and economic consequences for families and rural communities (*2*).

BU is characterized by a focal endemicity, and *M. ulcerans* has potential primary environmental reservoirs in wetlands, rivers, and stagnant bodies of water (*3*,*4*). The exact mode of transmission to humans remains unclear, but studies have shown that inoculation into the subcutaneous tissues is required (*5*,*6*). Thus, suspicions have arisen that aquatic insects, mollusks, and fishes are reservoirs and that insect bites are the mode of transmission (*7*–*9*). Transmission through human-to-human contact has been ruled out as a potential mode of transmission because living near an infected family member does not pose a higher risk for infection (*10*). However, fundamental questions remain concerning the participation of humans in dissemination of the bacterium (*11*,*12*).

Developing adapted preventive strategies requires identification of the environment that enables *M. ulcerans* development and the dynamics of the mycobacterium in the environment and in patients. However, because *M. ulcerans* cannot yet be cultured directly from environmental samples, comparison of *M. ulcerans* isolates retrieved in the environment with those in humans is impossible.

Whole-genome sequencing (WGS), coupled with single-nucleotide polymorphism (SNP)–based genotyping, has led to major advances in *M. ulcerans* genomics. This approach was applied recently to provide a description of the *M. ulcerans* population structure in Ghana (*13*). It has also been used to provide insights into the circulating genotypes in BU-endemic regions of Cameroon (*14*) and to study the evolution of *M. ulcerans* in Africa and southeastern Australia (*11*,*15*). Recently, Vandelannoote et al. described the bacterial distribution on a local scale in Congo (*12*).

In using a representative collection of 208 *M. ulcerans* isolates, our objective was to identify and track on a local scale the genotypes circulating in the BU-endemic regions of Ouémé and Plateau in southeast Benin and in Ogun State in southwest Nigeria. We evaluated the presence of specific clusters according to the geographic localization of patients and performed local-scale clustering by using a phylogenetic analysis approach based on SNP typing, coupled with spatial scan statistics.

## Materials and Methods

### Bacterial Isolates and Patients

We conducted WGS on 208 *M. ulcerans* strains isolated from patients diagnosed with and treated for BU during 2007–2016 at the Centre de Diagnostic et Traitement de la Lèpre et de l’Ulcère de Buruli (CDTLUB) in Pobè, Benin. We first sequenced and analyzed 179 strains; then, to perform validation of the model, we sequenced and analyzed a second set of 29 strains.

### DNA Sequencing

We cultivated isolates on a Lowenstein-Jensen medium for 5 months. We performed DNA extraction as previously described (*16*). We sequenced genomes by using either MiSeq or HiSeq sequencer (Illumina, https://www.illumina.com) with Nextrera XT DNA preparation kit or Ion Torrent S5XL technology with IonXpress Plus Fragment library kit (Life Technologies, https://www.thermofisher.com/us/en/home/brands/life-technologies.html). We submitted generated reads to the National Center for Biotechnology Information Sequence Read Archive (BioProjectID no. PRJNA499075). (See additional methods in Appendix 1)

### Variant Detection and Maximum-Likelihood Phylogenetics

After checking quality with FastQC version 0.11.7 (*17*), we performed a quality trimming by using Trimmomatic version 0.36 (*18*) and read mapping and SNP detection by using Snippy version 3.2 (*19*). We used the Burrows-Wheeler Aligner version 0.7.12 (*20*) with default parameters to map clipped read-pairs to the Agy99 reference genome (Genbank accession no. CP000325) and to the pMUM001 plasmid (accession no. BX649209) (*21*). Agy99 is the only annotated strain from Africa available for *M. ulcerans* species. By using the alignment of core genome SNPs of the first 179 genomes, we generated a maximum-likelihood phylogenetic tree with PhyML 3.020120412 using the general time-reversible model (*22*). For the second set of sequencing of 29 strains, we generated another tree by using the alignment of all the sequenced genomes. We performed bootstrapping by using 1,000 replicates to assess the reliability of the phylogenies. All phylogenies were rooted by using strains from Mu_A2 lineage. We used TreeCollapseCL 4.0 (*23*) to collapse nodes in the tree with bootstrap values below a set threshold of 70% to soft polytomies, thereby preserving the length of the tree.

### Phylogeographic Analysis

We performed a Kulldorf spatial scan statistic, implemented in SaTScan 9.6 (*24*), to verify the presence and location of spatial clusters by identifying spatial clusters on the basis of geographic coordinates (Appendix 1). We used QGIS 2.10 (*25*) to generate figures on the geographic distribution of *M. ulcerans*.

### Satellite Data and Processing

We acquired satellite imagery by using Sentinel-2 (European Space Agency, https://www.esa.int). Images used were recorded on January 6, 2018, with a spatial resolution of 10 m. Land use and cover were recovered with a supervised classification using minimum distance algorithm. We created training samples on the basis of expert knowledge of West Africa topography and Google Earth.

### Statistical Analysis

We tested for statistical significance by using either Fisher exact test or 1-way analysis of variance. We validated the model by calculating accuracy and Matthews correlation coefficient on the confusion matrices (Appendix 1). We performed data analysis and visualization in R 3.4.4 (*26*) with the ade4, plot3D, seqinr, and ggplot2 packages and data-intensive computations by using a GenOuest computer cluster (https://www.genouest.org).

## Results

### Presentation of Selected Strains and Areas

**Figure 1:**
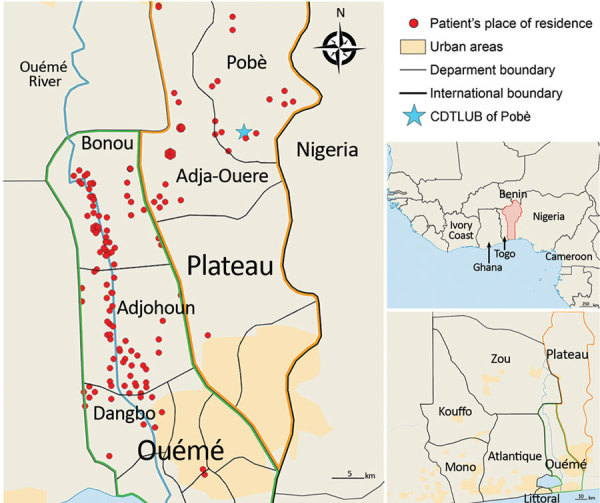
Spatial distribution of Buruli ulcer patients in Benin and Nigeria. The 179 sequenced genomes of *Mycobacterium ulcerans* were isolated from patients in southeastern Benin; 62% came from the Ouémé region, 26% came from the Plateau region, and the remaining genomes originated from patients in Nigeria. Red dots indicate precise locations of patients’ declared place of residence. In cases where several patients were from the same village, points were slightly displaced in a circle fashion to obtain the most accurate rendering of geographic density of Buruli ulcer cases. Insets show location of Benin in West Africa and of the Ouémé and Plateau regions in Benin. CDTLUB, Centre de Diagnostic et Traitement de la Lèpre et de l’Ulcère de Buruli.

We analyzed 179 strains isolated from patients diagnosed with and treated for BU at CDTLUB during 2007–2016. Patients originated from regions of Ouémé (111 [52%]) and Plateau (47 [26%]) in southeast Benin and from Ogun State in southwest Nigeria (21 [12%]). The proportion of genomes selected was in accordance with the geographic origin of patients visiting CDTLUB (Figure 1). Moreover, the 158 bacterial strains selected from Benin (Ouémé and Plateau) were representative of the epidemiologic data of BU in patients in Benin (Appendix 2 Tables 1). 

### Genome Sequence Comparisons of 179 *M. ulcerans* Strains

#### SNP Identification on *M. ulcerans* Strains

After WGS, a total of 6,163 core genome SNPs were uncovered after mapping the 179 strains against the referent genome Agy99 (Appendix 2 Table 3a); 35 SNPS (0.5%) were nonsense mutations, 2,544 (41.2%) were missense mutations, 1,539 (25%) were synonymous mutations, and 2,045 (33.2%) were outside of genes. Among these SNPs, 85% (5,223) belonged to 5 isolates identified as coming from Mu_A2 lineage and thus were used as a tree rooting outgroup. The 174 other isolates belonged to the West Africa lineage Mu_A1, and their genomes displayed highly restricted intrastrain genetic variation, having 940 SNP differences across a 5.2 Mbp core genome. Among these 940 SNPs (Appendix 2 Table 3b), 9 (1%) were nonsense, 398 (42%) were missense, 228 were synonymous (24%), and 305 (33%) were outside of genes. Also, although the plasmid accounted for 3.1% of the total amount of the bacterial genome, only 9 SNPs (0.9%) were found, none occurring on genes that encoded enzymes required for mycolactone synthesis. Thus, most (99%) SNPs were located on the bacterial chromosome. 

#### Identification of 8 Genetically Distinct *M. ulcerans* Genotypes

**Figure 2:**
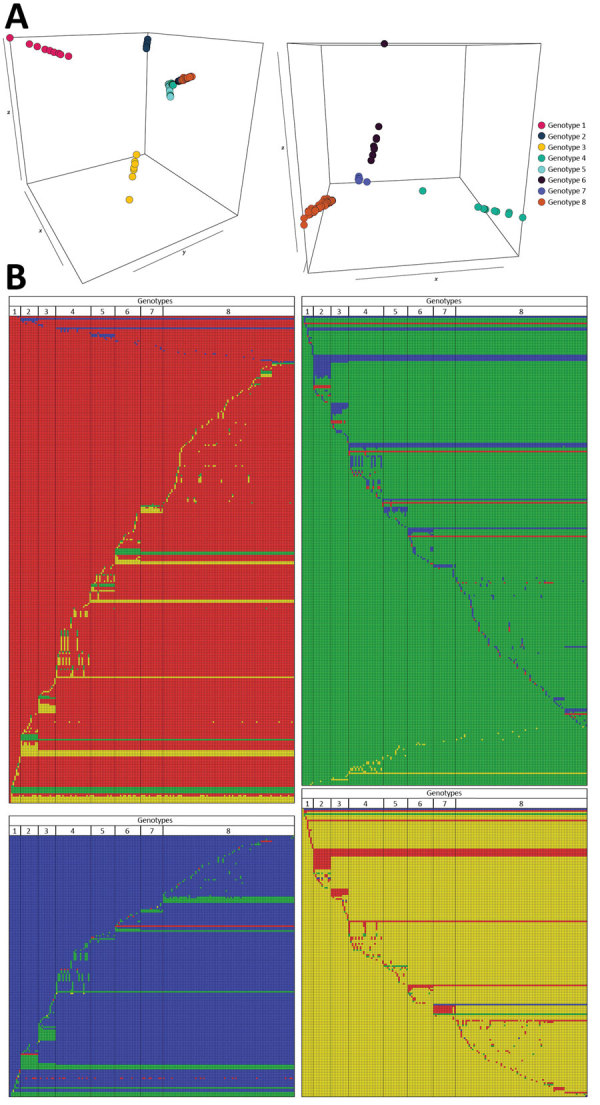
Graphical representations of the 8 *Mycobacterium ulcerans* genomes and their 940 single-nucleotide polymorphisms from Buruli ulcer patients in Benin and Nigeria. A) Principal component analysis (PCA) projection on the first 3 principal components with 8 groups of genomes clustering together, which we defined as genotypes. PCA was performed based on the Eigenstrat algorithm but applied to a haploid organism. Image on the left displays a PCA performed on all 174 genomes; image on the right displays a PCA performed after removing genomes from the first 3 genotypes (shown for better visualization of genome clustering). Axes x, y, and z represent the principal components 1, 2, and 3, respectively; inertia was 7% for component 1, 5% for component 2, and 4% for component 3. B) Graphical representation of the 940 single-nucleotide polymorphisms specific to the 8 genotypes, showing interdifferences and intradifferences of all genomes. Each line represents 1 genomic position, and each column represents 1 *M. ulcerans* genome. A color code has been chosen for each nucleotide (blue, adenine; green, guanine; red, cytosine; yellow, thymine). Each representation has been ordered and referenced against the genome 1232–13 belonging to genotype 1 (first column).

We used an Eigenstrat-like principal component analysis approach to identify groups of genomes based on their SNP. We identified 8 groups with similar genotypic features and defined them as genotypes (Figure 2, panel A). We displayed the 940 SNPs at each genomic position (Figure 2, panel B).

#### Phylogenetic Inference of the 8 Genetically Distinct *M. ulcerans* Genotypes

**Figure 3:**
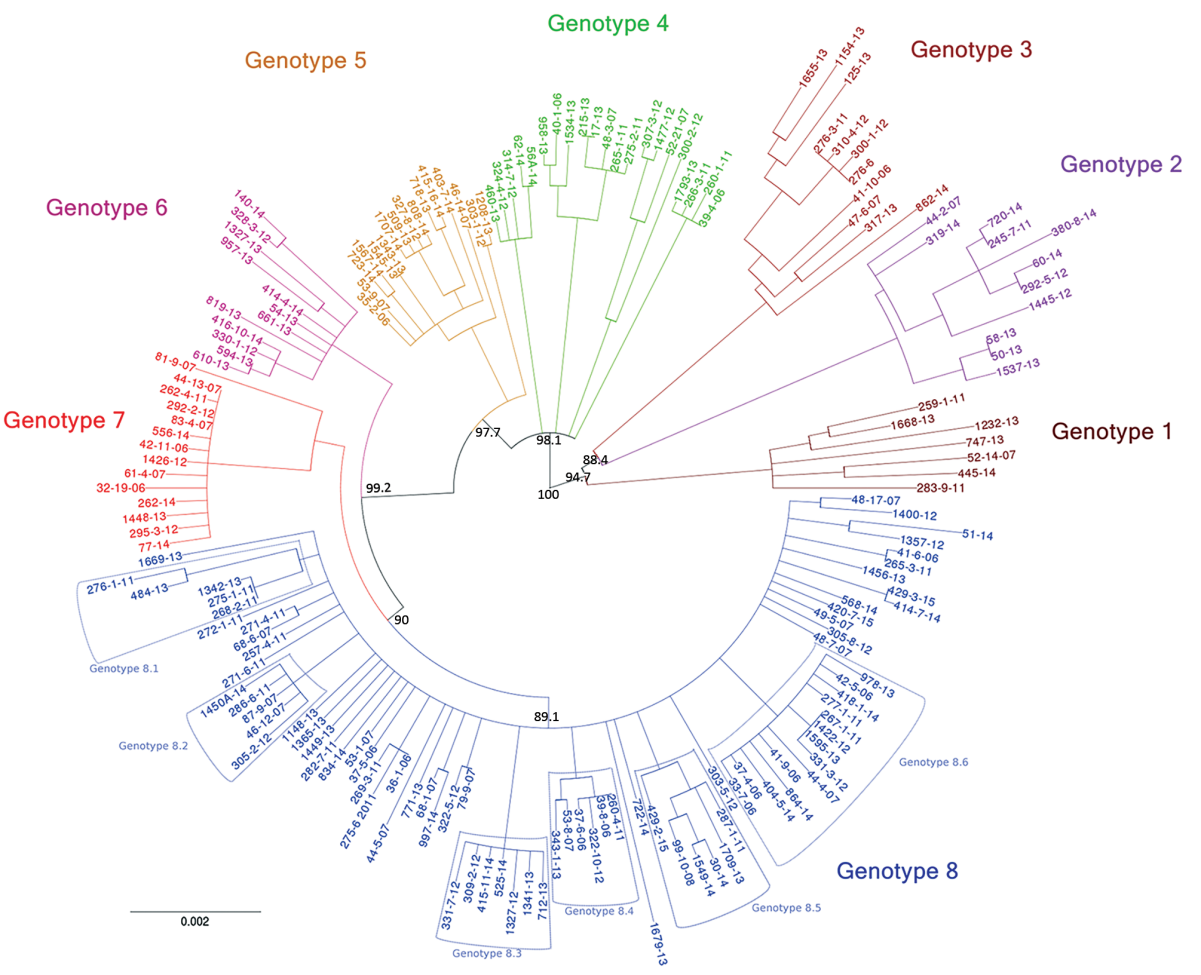
Eight genotypes emerging from phylogenetic analysis of *Mycobacterium ulcerans* isolates from Buruli ulcer patients in Benin and Nigeria. This rooted circular phylogenetic tree was built by using PhyML (*22*) on the basis of the core alignment of all single-nucleotide polymorphisms obtained with Snippy 3.2 (*19*). The bootstrap values are only represented on primitive branches. Branches with bootstrap values <70% were collapsed as polytomies. The outgroup (Papua New Guinea genomes) and the reference genome (Agy99) are not represented (see Appendix 1 Figure 1). On the basis of the segregation indicated by this tree, the genomes were divided in 8 genotypes, which are either monophyletic or paraphyletic. Each taxon was assigned a specific color. Subgenotypes of genotype 8 also are indicated. Scale bar indicates the Nei genetic distance.

The 8 genotypes were also identifiable in the phylogeny of the 174 strains identified as belonging to Mu_A1 lineage (West Africa lineage) (Figure 3; Appendix 1 Figure 1). Almost half (46%) of the strains belonged to genotype 8; the rest belonged to genotypes 1–7 at proportions ranging from 4% to 12% (Figure 2). Each genotype seemed to be a monophyletic group, with the exception of genotypes 4 and 5, which were paraphyletic. Each group had a bootstrap value ranging from 88% to 100%. Therefore, we proposed a mutation profile for each genotype, thereby providing a specific molecular signature as a basis for bacterial strain genotyping (Appendix 1 Figure 2). We compiled each SNP specific relationship to a genotype (Appendix 2 Table 3).

### Effect of Genotype Specificity on Clinical Features

**Figure 4:**
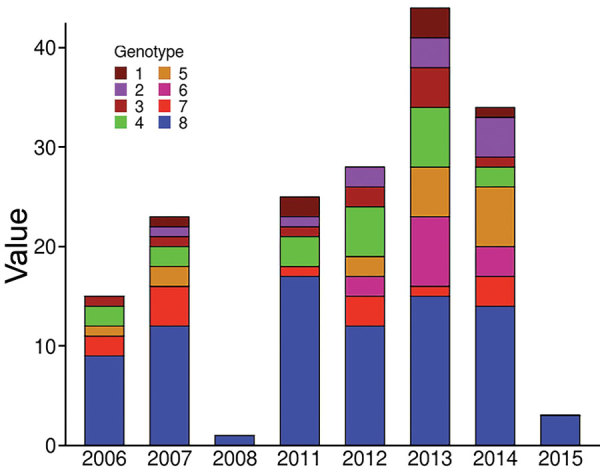
Distribution of *Mycobacterium ulcerans* genotypes according to diagnosis date for Buruli ulcer patients in Benin and Nigeria. The distribution of genotypes was tested on 2 × 8 contingency tables (Fisher exact test) to compare each year to one another.

To verify whether the 8 genotypes could be related to any of the basic characteristics of patients, we performed the Fisher exact test to analyze severity and sex and analysis of variance to analyze age. We found no significant association regarding severity, sex, or age (data not shown). We also considered finding an association between genotypes and presence of bone lesions. Our results showed no association between genotype and higher or lower incidence of osteomyelitis (data not shown). However, this lack of finding could be attributable to the limited amount of reported bone damage in our sampling (only 4 cases). We found no association between genotype and the year of strain isolation (Figure 4).

### Identification of Spatial Clusters in Benin

**Figure 5:**
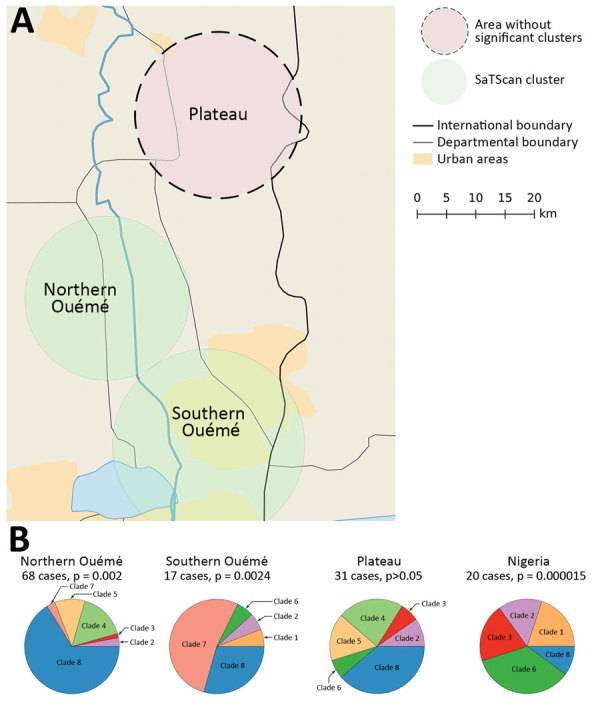
Spatial cluster detection results of *Mycobacterium ulcerans* genotypes for Buruli ulcer patients in Benin and Nigeria. A) Two significant areas detected along the Ouémé River. Three regions of interest are shown on the map. Two (northern Ouémé and southern Ouémé) show significant spatial clustering of genotypes; a nonsignificant area (Plateau) is given for reference. B) Composition of these 3 clusters, compared with the composition that would be expected from a random distribution.

To examine the relationship between phylogenetic classification and *M. ulcerans* geographic origin, we used a multinomial spatial scan statistic (Appendix 1). We found a first significant cluster (p = 0.002), with a radius of 15.7 km^2^, that included 68 cases and was located in northern Ouémé (Figure 5). This cluster contained strains belonging mainly to genotypes 4 and 8; relative risk (RR) for infection was 1.5 for genotype 4 and 1.9 for genotype 8 (Table 1). In contrast, within this area, the RR for infection with a strain of genotype 2, 3, 5, and 7 was low (RR 0.6, 0.2, 1.0, and 0.4, respectively), and the RR for infection with a strain belonging to genotype 1 or 6 was null. The second significant cluster (p = 0.0024) was located in southern Ouémé, with a radius of 18.8 km^2^, and included 17 strains. The most notable feature of this cluster was the high risk for infection with a strain of genotype 7 (Table 1). Indeed, a patient with BU living in this area was 20 times more likely to have been infected with this genotype than a BU person living outside this area. Surprisingly, the multinomial spatial scan statistic did not identify any significant cluster in Plateau, meaning that strains in Plateau are similar to a random distribution of all *M. ulcerans* genotypes (Figure 5). These data suggest a difference in bacterial life cycle between Ouémé and Plateau in terms of bacterial persistence.

**Table 1:** Cluster detection analysis for predominance of *Mycobacterium ulcerans* genotypes using the maximum reported spatial window of 50% of the sample population and an univariate scan statistic, Benin and Nigeria*

Spatial cluster	Radius, km^2^	LLR	Ob	Genotype

1		2		3		4		5		6		7		8
RR (%)	RR (%)	RR (%)	RR (%)	RR (%)	RR (%)	RR (%)	RR (%)
Northern Ouémé	15.7	19.2	68	0		0.6 (4.1)		0.2 (1.3)		1.5 (15)		1 (9.6)		0		0.4 (4.1)		1.9 (66)
Southern Ouémé	17.9	18.8	17	1.5 (5)		1 (6)		0		0		0		0.9 (6)		20 (53)		0.6 (29)
Nigeria†	54.3	27.1	20	9.9 (20)		3.2 (15)		5 (20)		0		0		13 (35)		0		0.2 (10)

*Percentages indicate falling in each group within a cluster. LLR: log likelihood ratio; Ob, number of observations in a cluster; RR, relative risk, computed as the ratio of the proportions of the number of Buruli ulcer cases in each category out of the total number of cases inside the cluster versus outside. †Ogun State.

### Identification and Distribution of Genetic Subgroups Belonging to Genotype 8

**Figure 6:**
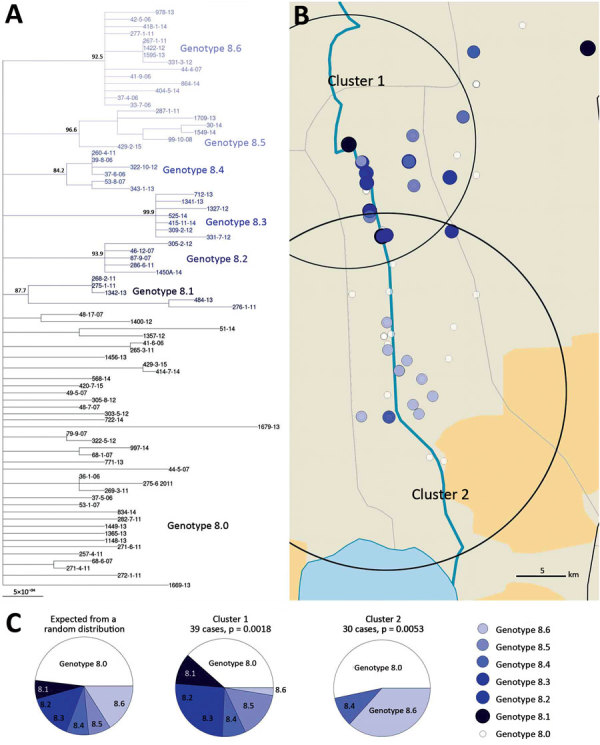
Spatial cluster detection results of *Mycobacterium ulcerans* genotype 8 for Buruli ulcer patients in Benin and Nigeria. Genotype 8 population was identified in 2 clusters along the Ouémé River. A) Phylogenetic tree of genotype 8 reveals the presence of potentially emerging genotypes. B) Location of the clusters. The analysis shows 2 significant clusters where specific subgenotypes are overrepresented compared to outside of these areas. C) Composition of these 2 clusters compared with the composition that would be expected from a random distribution.

Among the 8 genotypes identified from the West Africa lineage, genotype 8 held almost half of the *M. ulcerans* strains and was present in Ouémé and Plateau. We focused on this genotype and found 6 subgenotypes phylogenetically distinguished, with well-supported nodes and bootstrap values ranging from 84% to 99% (Figures 3, 6; Appendix 2 Table 3). Subgenotypes 8.1 to 8.6 contained half of the total strains belonging to genotype 8. The other half was assigned to the denomination 8.0 as not belonging to a particular subgenotype. Because genotype 8 was found mainly near the Ouémé River, we assessed the distribution of subgenotypes in this area by using a spatial scan statistic similar to that we described previously. We found 2 statistically significant clusters along the Ouémé River, 1 in the north (cluster 1) and 1 in the south (cluster 2) (Figure 6). Subgenotypes 8.1, 8.2, 8.3, and 8.5 were found only in cluster 1 (i.e., in the north (Table 2). Subgenotypes 8.1 and 8.3 had higher RRs (4.31 and 2.69, respectively) within this area compared with outside the area. The risk for carrying subgenotype 8.6 strains within this cluster was significantly low (RR 0.1) (Table 2). However, we found only 2 subgenotypes (8.4 and 8.6) in cluster 2 (RR 9.4 for subgenotype 8.6 and 1.7 for subgenotype 8.4) (Table 2). Clusters 1 and 2 (Figure 6) were slightly displaced compared with the northern and southern clusters (Figure 5) but overlapped considerably.

**Table 2:** Cluster detection analysis for predominance of subgenotypes of *Mycobacterium ulcerans* genotype 8, Benin*

Spatial cluster	Radius, km^2^	LLR	Ob	Subgenotype												
				8.0		8.1		8.2		8.3		8.4		8.5		8.6
				RR (%)		RR (%)		RR (%)		RR (%)		RR (%)		RR (%)		RR (%)
Cluster 1	14.7	15.7	39	0.7 (38.5)		4.3 (10.3)		Inf (12.8)		2.69 (12.8)		1.08 (7.7)		Inf (15.4)		0.1 (2.6)
Cluster 2	20.0	17.3	30	1.2 (53.3)		0 (0)		0 (0)		0 (0)		1.7 (10)		0 (0)		9.4 (36.7)

*Percentages indicate falling in each group within a cluster. Inf, infinite; LLR: log likelihood ratio; Ob, number of observations in a cluster; RR, relative risk, computed as the ratio of the proportions of the number of Buruli ulcer cases in each category out of the total number of cases inside the cluster versus outside.

### Land Cover and Genotype Distribution

**Figure 7:**
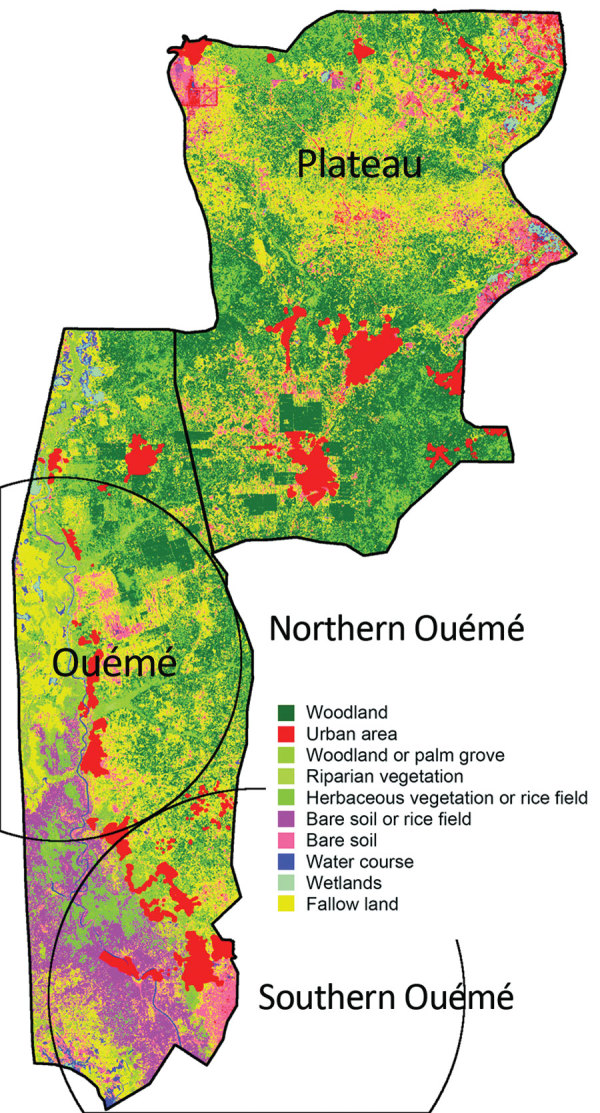
Land use and land cover assessment from Sentinel-2 imaging of Benin. The Ouémé region has specific land and plant formations, such as grassy savanna, grasslands, and swamps. Soils easily become saturated with water because of a shallow water table and the proximity of a river, which causes floods and a natural delta formation in the south of the region. Circles indicate the detected northern and southern Ouémé *Mycobacterium ulcerans* clusters.

Genotypes were not distributed randomly between southern and northern Ouémé, suggesting that the clusters might be associated with specific land cover. Globally, the 2 regions differed significantly in terms of land use and cover, and a high soil heterogeneity existed between the north and south (Figure 7). Whereas the south has flooded soils suitable for market gardening and marshes for tree cultivation, the north mainly consists of forests and palm groves, and agricultural land is sparse. This difference might indicate that *M. ulcerans* strains of genotype 7 will most likely be found in bare soils and rice fields, whereas *M. ulcerans* strains of genotype 8 will most likely be found in areas with riparian vegetation, herbaceous vegetation, and woodlands.

### Specificity of the Nigeria Strains

**Figure 8:**
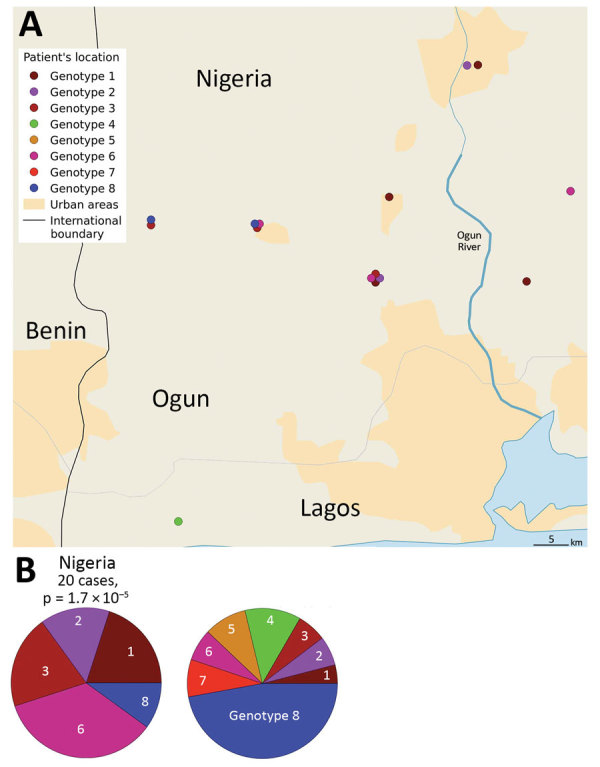
Difference in *Mycobacterium ulcerans* genotype distribution between Ogun State (Nigeria) and Benin. A) Locations of patients and the genotypes of the strains. B) Composition of the cluster in the Ogun area compared with the composition that would be expected from a random distribution. The genotype distribution of the Ogun was fundamentally different from those in Benin.

The CDTLUB in Pobè is located near the Nigeria–Benin border, and several BU patients from Nigeria were treated in the center. Of the 179 strains sequenced in our study, 21 were isolated from patients from Ogun State in southwestern Nigeria. This area is dependent on a different drainage basin than that used by Ouémé, thus providing an opportunity to study *M. ulcerans* diversity and distribution in another independent BU-endemic area. Spatial analysis showed that the most significant cluster (p<0.0001) covered the area of Ogun State. This cluster was drastically different from those found in the Ouémé region of Benin. In this region of Nigeria, the RRs for infection with strains from genotype 1, 2, 3, or 6 were significantly higher than for any other genotype (Figure 8). The RR for infection with these genotypes was 9.9 times higher for genotype 1, 3.2 times higher for genotype 2, 5 times higher for genotype 3, and 13 times higher for genotype 6 in this area compared with outside the area (Table 1). Furthermore, the RR for infection with genotype 8 was negligible (0.2), even though it was the most widespread genotype along the Ouémé River (Table 1). We observed a similar nonrandom distribution in Ouémé and in Ogun State.

### Validation of Multinomial Model Demonstrating Geographic Clusters

To validate the distribution of genotypes in both BU-endemic areas, we developed a spatial model of genotype dispersion on the basis of phylogenetic classification. To verify the power of our model to correctly associate a genotype to a genome given its geographic origin, we performed WGS on 29 additional bacterial strains. These strains were isolated from patients at CDTLUB who had been diagnosed with BU during 2015–2017 and were living in northern Ouémé (11 patients), southern Ouémé (9 patients), Plateau (4 patients), and Nigeria (5 patients). We identified 2 of the 29 strains as part of the MU_A2 lineage and added them to the outgroup to build a new phylogenetic tree. The 27 other strains were easily included in the phylogenetic tree (Figure 2) without altering the classification of the 8 different genotypes or the cluster classifications. Plateau contained no statistically significant clusters.

For each of the 3 significant geographic clusters, we compared the observed repartition of new genomes to the expected distribution given by each cluster. The 2 clusters in Ouémé exhibited strong accuracy (91%) and a Matthews coefficient >0.6, corresponding to a strong relationship (Table 3). The Nigeria cluster model showed lower accuracy (85%) and a Matthews coefficient of 0.371, corresponding to a moderately positive relationship. These results support the existence of a spatial cluster of *M. ulcerans* genotypes in some BU-endemic areas.

**Table 3:** Statistical measurements of the performance of the multinomial models on a real dataset of newly sequenced genomes, Benin and Nigeria*

Locale	Accuracy†	Matthews correlation coefficient‡
Northern Ouémé	91.44%	0.608*
Southern Ouémé	91.73%	0.637*
Nigeria (Ogun State)	86.25%	0.371**

†Sum of the true positive and true negative divided by the total population. ‡Contingency matrix method of calculating the Pearson product–moment correlation coefficient (*27*). Its value ranges from −1 to +1 with +1 being a perfect positive prediction model, 0 no better than random prediction, and −1 being a model where predicted class is in total contradiction with observed class. Values indicated the strength of the relationship (*, strong positive relationship; **, moderate positive relationship).

## Discussion

BU occurs in poor rural communities with little economic or political influence. A key epidemiologic feature of this disease is the distribution of cases in very well-delimited foci. However, in these areas, the precise zones of high-risk contamination in environments are not identified. As with other neglected tropical diseases, fighting BU will require integrated approaches to reduce transmission of the causative mycobacterium and ensure earlier patient management.

Socioeconomic factors, environmental changes, ecologic factors, and the conquest of new territories promote infections caused by pathogens with a wildlife origin (*28*–*30*). In the field of BU, all epidemiologic studies show that environmental changes, particularly wetland creation, deforestation, and socioeconomic factors that promote contact with nonprotected water, enhance the spread of the disease (*3*,*28*,*31*–*37*). Although all epidemiologic and environmental studies underline the main role of ecologic factors in *M. ulcerans* transmission, the precise route of *M. ulcerans* transmission to humans remains unclear. Molecular epidemiology studies conducted on a local scale can be adapted to elucidate the structure, diversity, evolution, dissemination, and life of the bacterial population.

The genome of *M. ulcerans* consists of a main chromosome and a giant plasmid containing the gene encoding for enzymes synthesizing the mycolactone. Because this genome has low variation, conventional genetic methods can only differentiate isolates on a continental scale (*38*). WGS offers a much greater resolution and could be used for studying *M. ulcerans* diversity on a local scale by analyzing SNPs (*11*). SNP analysis of our 174 *M. ulcerans* isolates belonging to the West Africa lineage Mu_A1 enabled us to identify 8 genotypes on the basis of 940 SNP positions. This analysis revealed a high conservation, especially on plasmid sequences, highlighting the crucial role of mycolactone toxin to colonize specific environmental niches, including human (*39*,*40*). The main role of mycolactone in host colonization was affirmed because no link could be established between this genomic diversity and clinical manifestations. Furthermore, the distribution of gene mutations based on a functional annotation is similar to the distribution of all the classified genes of *M. ulcerans* (Appendix 1 Figure 3), supporting the hypothesis that acquisition of a mutation has no relation to its ability to colonize a host or with its virulence.

The particularity of our study was the spatial local-scale analysis of the isolates. We used a phylogenetic analysis approach based on SNP-typing, coupled with spatial scan statistics. This method is more suitable for working in a well-defined BU-endemic area in a short period (a few years), whereas a Bayesian phylogenetic approach is suitable for studying temporal distribution of *M. ulcerans* over a much longer period (decades) (*11*,*15*).

Our spatial analysis revealed the existence of a geographic clustering of *M. ulcerans* genotypes in southeastern Benin and southwestern Nigeria. On this scale, our results showed a strong association between hydrologic drainage areas and *M. ulcerans* genotypes, because a clear difference was observed in the distribution of genotypes between BU patients living around Nigeria’s Yewa basin and Benin’s Ouémé basin. Our clustering revealed that bacteria evolved independently and differentially, depending on their specific ecologic reservoir. Moreover (and more surprisingly), we were able to detect clustering of *M. ulcerans* genotypes along a same drainage basin (in this case the Ouémé basin). Inside the main genotype (genotype 8), we were also able to detect subgenotypes with a similar clustering along the river, indicating dissemination of *M. ulcerans* on a local scale and then a persistence of *M. ulcerans* in independently endemic niches. These findings are consistent with previous scenarios in which *M. ulcerans*, once introduced into a new environment, expands instead of becoming a quiescent pathogen (*11*).

In considering the nature of the land cover, we observed striking heterogeneity along the river, pinpointing the compartmentalization of different environmental niches (Figure 7). On the other hand, the predominance of 1 genotype in 1 area associated with a particular land cover suggests that patients frequent the same type of contamination source, and the hypothesis that acquisition of infection is local has already been proposed (*11*). Altogether, our study gives a precise cartography of *M. ulcerans* genotype distribution, revealing a well delimited high-risk area where preventive strategies, active diagnosis, and epidemiologic surveillance must be focused.

Unlike the Ouémé region and southwestern Nigeria, the lack of any spatial cluster in the Plateau region suggests differences in terms of dissemination and environmental persistence. Plateau separates the Ouémé and Yewa draining basins, and the bacterial genotypes in the Plateau area are a mix of the genotypes in these 2 basins. This signature could be explained by different hypotheses. First, there might be a contamination site different from the place of residence given that persons living on the Plateau might be contaminated in Ouémé or Nigeria during their travels. This hypothesis does not explain all contaminations because patients’ histories revealed that some of them had never left their village. Second, there might be a nonpersistent presence of *M. ulcerans* in the Plateau environment, in which *M. ulcerans* might be disseminated from Ouémé and Nigeria to Plateau by mammals (including humans) or flying insects and might be present in aquatic niches for only the few months when wetlands exist just after the rainy seasons (*41*). This situation rules out humans as the main carrier and reservoir of *M. ulcerans* and supports the position that humans with active infection are unlikely to play a major role in the bacterial ecology. Moreover, BU is known to be a locally acquired infection rather than an imported one (*42*). Thus, the most plausible hypothesis in the particular case of the Plateau region is based on the inability of the bacterium to develop for a period long enough in transitional aquatic reservoirs, thereby preventing the production of new genotypes. Treating humans against *M. ulcerans* infection might not be sufficient to break disease transmission chains as previously suggested (*12*).

Because only *M. ulcerans* strains isolated from patients were analyzed, it would have been interesting, as a next step, to compare diversity of *M. ulcerans* from humans and environmental samples to better understand the interactions between this pathogen and the host. Combined with the tools we developed to reveal the genetic diversity of *M. ulcerans* in humans, DNA enrichment techniques, such those reported in studies on *Borrelia* spp., could be improved to meet this challenge (*43*). In conclusion, our approach allowed the identification of delimited high-risk contamination areas, paving a new avenue to develop prevention and intervention strategies.

Appendix 1 Additional information (detailed methods and figures) regarding stable and local reservoirs of *Mycobacterium ulcerans* inferred from the nonrandom distribution of bacterial genotypes, Benin.

Appendix 2 Additional information (tables) regarding stable and local reservoirs of *Mycobacterium ulcerans* inferred from the nonrandom distribution of bacterial genotypes, Benin.
