# Supplementary material for: Stable and Local Reservoirs of Mycobacterium ulcerans Inferred from the Nonrandom Distribution of Bacterial Genotypes, Benin
Source: Emerg Infect Dis. 2020 Mar;26(3):491–503. doi: 10.3201/eid2603.190573 (PMC7045821; doi:10.3201/eid2603.190573)
Supplement: Appendix 1 — Additional information (detailed methods and figures) regarding stable and local reservoirs of Mycobacterium ulcerans inferred from the nonrandom distribution of bacterial genotypes, Benin. [file 19-0573-Techapp-s1.pdf]

Article DOI: https://doi.org/10.3201/eid2603.190573

Stable and Local Reservoirs of *Mycobacterium ulcerans* Inferred from the Nonrandom Distribution of Bacterial Genotypes, Benin

Appendix 1

Supplementary Methods

Bacterial Isolates and Patients

This study was performed by working closely with the CDTLUB of Pobè. This specialized hospital has been actively monitoring the disease since 2006 and has collected substantial epidemiologic data (age, sex, village/city of residence, etc.). Indeed, between 2006 and 2015, the CDTLUB of Pobe diagnosed and treated 1761 PCR-confirmed Buruli ulcer cases coming from Oueme (63%), Plateau (28%) and Nigeria (9%).

Genome Sequencing

DNA sequencing of 179 samples was undertaken using either an Illumina MiSeq or HiSeq with Nextera XT DNA preparation kit (Illumina, San Diego, California) and 29 strains was performed using Ion Torrent S5XL with IonXpress Plus Fragment Library kit (Life Technologies, California). The quality of raw reads was assessed using FastQC v0.11.7 (*1*). DNA sequences have been submitted to the public Sequence Read Archive (SRA) -NCBI open access database. All genomes possessed a mean sequencing coverage superior to 30 reads and less than 20% of the bacterial chromosome was covered by less than 15 reads for efficient variant discovery. Prior to further analysis, reads were cleaned using Trimmomatic v0.36 (*2*). Reads were filtered to remove potentials adapters, ambiguous and unidentified base calls or any reads inferior to 35bp as well as portion of reads whose average quality drops below 15 on a 4-base wide sliding window. During mapping of reads on the reference genome, the repetitive and ubiquitous IS2404 and IS2606 genomic elements were hard-masked and consequently eliminated because of the unreliability of mapping on repetitive and mobile regions.

Multinomial Spatial Scan Statistic

The method uses a circular window of varying radius centers at each location that moves across the map so that, at any given position, the window includes different sets of neighboring residencies. At each position, the radius of the circular window varies repeatedly from zero up to a predefined maximum radius, corresponding to a window including 50% of the total study population. This method allows the circular window to continuously vary in both location and size, thereby creating a large number of distinct potential clusters. The detection of clusters was performed by comparing the number of cases within the window with the number expected if cases were randomly distributed in space, using a multinomial model (*3*). The unit of space was defined by the coordinates of the patient's villages. Clusters were scanned where specific genotypes of *M. ulcerans* were more or less prominent, i.e., areas where the observed number of cases differs from the expected number of cases. The significance of the identified clusters was tested with a likelihood ratio test and statistical inference for the spatial clusters was evaluated based on a Monte Carlo hypothesis test with 999 replications.

Statistical Analysis and Performance Metrics

Comparisons of the expected genotype distribution inside each statistically significant clusters found in SatScan with the genotype distribution of the second set of sequencing were performed by calculating accuracy and Matthews correlation coefficient on confusion matrices. Accuracy measures the fraction of all instances that are correctly categorized while Matthews correlation coefficient summarizes the overall correlation like the Pearson correlation coefficient (*4*) and thus possesses the same interpretation (*5*).

References

1. Andrews S. FastQC: a quality control tool for high throughput sequence data. 2010 [cited 2019 Apr 1]. https://www.bioinformatics.babraham.ac.uk/projects/fastqc

2. Bolger AM, Lohse M, Usadel B. Trimmomatic: a flexible trimmer for Illumina sequence data. Bioinformatics. 2014;30:2114–20. PubMed https://doi.org/10.1093/bioinformatics/btu170

3. Jung I, Kulldorff M, Richard OJ. A spatial scan statistic for multinomial data. Stat Med. 2010;29:1910–8. PubMed https://doi.org/10.1002/sim.3951

4. Matthews BW. Comparison of the predicted and observed secondary structure of T4 phage lysozyme. Biochim Biophys Acta. 1975;405:442–51. PubMed https://doi.org/10.1016/0005-2795(75)90109-9

5. Powers DMW. Evaluation: from precision, recall and F-Measure to ROC, informedness, markedness, and correlation. J Mach Learn Technol. 2011;2:37–63.

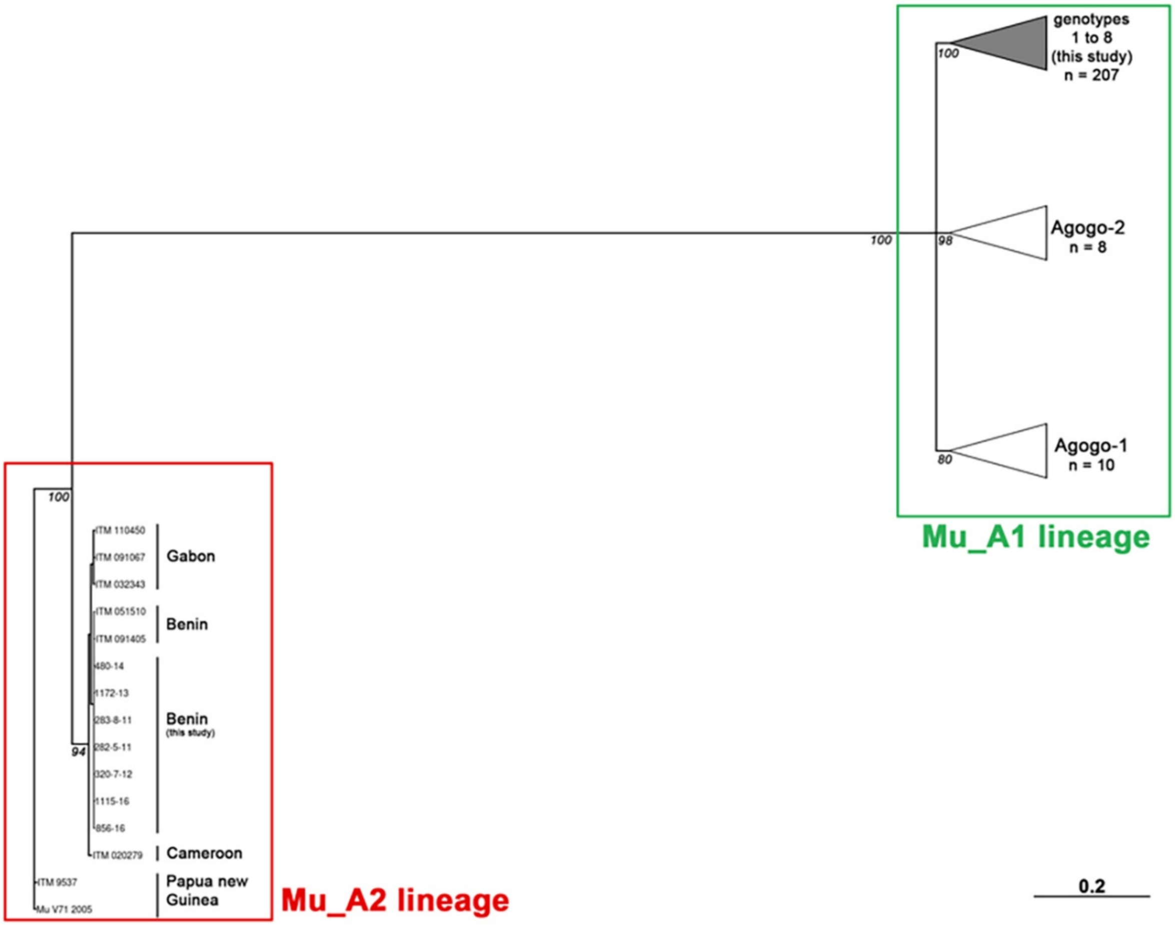

Appendix 1 Figure 1. Phylogenetic tree showing the relationship between the genomes from our study and genomes obtained from two other studies performed in central and West Africa. Genomes from the Mu_A2 lineage were obtained from the NCBI Sequence Read Archive, accessible under the BioProject accession PRJNA313185, declares country of origin is precised for each genome. The genomes from the Agogo lineages were obtained from the BioProject PRJEB8235 and are all coming from Ghana.

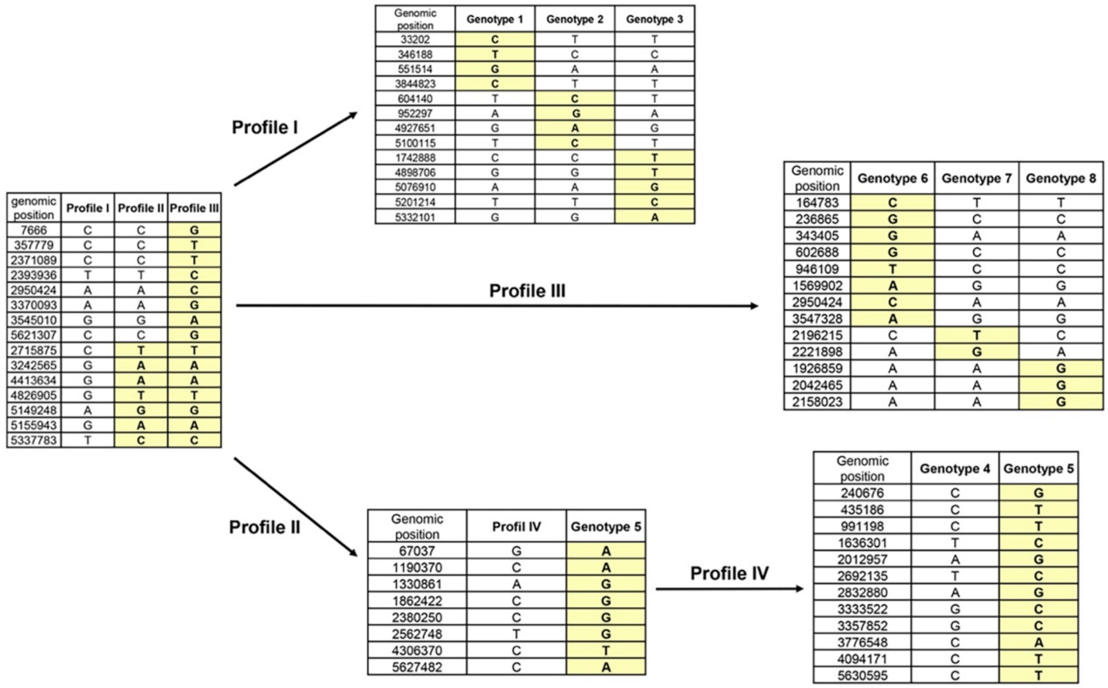

genomic position	Profile I	Profile II	Profile III
7666	C	C	G
357779	C	C	T
2371089	C	C	T
2393936	T	T	C
2950424	A	A	C
3370093	A	A	G
3545010	G	G	A
5621307	C	C	G
2715875	C	T	T
3242565	G	A	A
4413634	G	A	A
4826905	G	T	T
5149248	A	G	G
5155943	G	A	A
5337783	T	C	C

Genomic position	Genotype 1	Genotype 2	Genotype 3
33202	C	T	T
346188	T	C	C
551514	G	A	A
3844823	C	T	T
604140	T	C	T
952297	A	G	A
4927651	G	A	G
5100115	T	C	T
1742888	C	C	T
4898706	G	G	T
5076910	A	A	G
5201214	T	T	C
5332101	G	G	A

Genomic position	Genotype 6	Genotype 7	Genotype 8
164783	C	T	T
236865	G	C	C
343405	G	A	A
602688	G	C	C
946109	T	C	C
1569902	A	G	G
2950424	C	A	A
3547328	A	G	G
2196215	C	T	C
2221898	A	G	A
1926859	A	A	G
2042465	A	A	G
2158023	A	A	G

Genomic position	Profil IV	Genotype 5
67037	G	A
1190370	C	A
1330861	A	G
1862422	C	G
2380250	C	G
2562748	T	G
4306370	C	T
5627482	C	A

Genomic position	Genotype 4	Genotype 5
240676	C	G
435186	C	T
991198	C	T
1636301	T	C
2012957	A	G
2692135	T	C
2832880	A	G
3333522	G	C
3357852	G	C
3776548	C	A
4094171	C	T
5630595	C	T

Appendix 1 Figure 2. Flowchart of genotyping strategy with their specific genomic variant. This chart provides a comprehensive method based on a specific number of genomic position to use to assign a strain to its genotype. Nucleotide substitutions for genotypes are highlighted in yellow.

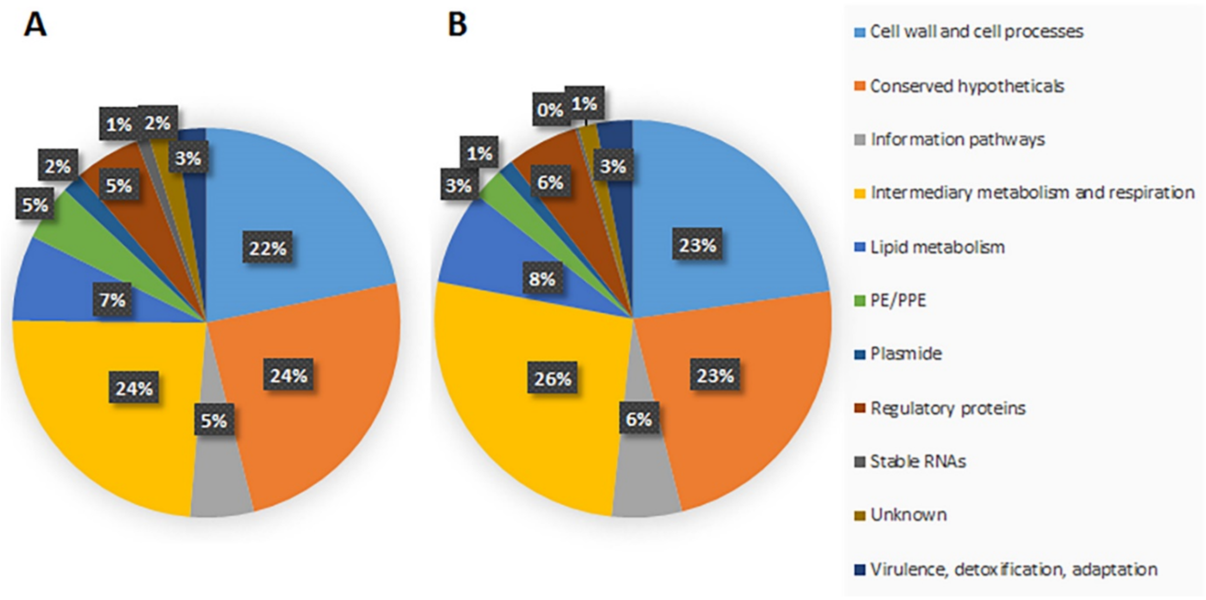

Appendix 1 Figure 3. Gene distribution based on functional annotation. A) Distribution of all annotated genes (4771) by BURUlist of *M. ulcerans*. Each gene belongs to one of the functional categories. B) Distribution of genes with at least one SNP (1173) in each functional category.
